# Effects of High-Intensity Interval Training versus Continuous Training on Physical Fitness, Cardiovascular Function and Quality of Life in Heart Failure Patients

**DOI:** 10.1371/journal.pone.0141256

**Published:** 2015-10-30

**Authors:** Nathalie M. M. Benda, Joost P. H. Seeger, Guus G. C. F. Stevens, Bregina T. P. Hijmans-Kersten, Arie P. J. van Dijk, Louise Bellersen, Evert J. P. Lamfers, Maria T. E. Hopman, Dick H. J. Thijssen

**Affiliations:** 1 Radboud university medical center, Radboud Institute for Health Sciences, Department of Physiology, Nijmegen, the Netherlands; 2 Radboud university medical center, Radboud Institute for Health Sciences, Department of Cardiology, Nijmegen, the Netherlands; 3 Canisius-Wilhelmina Hospital, Department of Cardiology, Nijmegen, the Netherlands; 4 Liverpool John Moores University, Research Institute for Sport and Exercise Sciences, Liverpool, United Kingdom; Tokai University, JAPAN

## Abstract

**Introduction:**

Physical fitness is an important prognostic factor in heart failure (HF). To improve fitness, different types of exercise have been explored, with recent focus on high-intensity interval training (HIT). We comprehensively compared effects of HIT *versus* continuous training (CT) in HF patients NYHA II-III on physical fitness, cardiovascular function and structure, and quality of life, and hypothesize that HIT leads to superior improvements compared to CT.

**Methods:**

Twenty HF patients (male:female 19:1, 64±8 yrs, ejection fraction 38±6%) were allocated to 12-weeks of HIT (10*1-minute at 90% maximal workload—alternated by 2.5 minutes at 30% maximal workload) or CT (30 minutes at 60–75% of maximal workload). Before and after intervention, we examined physical fitness (incremental cycling test), cardiac function and structure (echocardiography), vascular function and structure (ultrasound) and quality of life (SF-36, Minnesota living with HF questionnaire (MLHFQ)).

**Results:**

Training improved maximal workload, peak oxygen uptake (VO_2peak_) related to the predicted VO_2peak_, oxygen uptake at the anaerobic threshold, and maximal oxygen pulse (all *P*<0.05), whilst no differences were present between HIT and CT (N.S.). We found no major changes in resting cardiovascular function and structure. SF-36 physical function score improved after training (*P*<0.05), whilst SF-36 total score and MLHFQ did not change after training (N.S.).

**Conclusion:**

Training induced significant improvements in parameters of physical fitness, although no evidence for superiority of HIT over CT was demonstrated. No major effect of training was found on cardiovascular structure and function or quality of life in HF patients NYHA II-III.

**Trial Registration:**

Nederlands Trial Register NTR3671

## Introduction

Heart failure (HF) is a common disease with an increasing prevalence worldwide [1], and is characterized by a low 5-year survival of 35–55% [2–4]. Several studies have indicated that physical fitness is an important prognostic factor for HF patients, in which a low physical fitness is associated with higher mortality rates [5,6]. Previous studies demonstrate that exercise training can improve physical fitness [7], cardiac function [8], vascular function [9,10], and quality of life [7] in HF patients [11]. Therefore, exercise training is recommended for HF patients and encompasses a central component of cardiac rehabilitation [12].

Most previous studies examining the impact of exercise training in HF patients have adopted exercise at moderate-intensity. Recent studies explored the impact of high-intensity interval training (HIT), which can be described as short periods of exercise performed at high-intensity (>80–85% peak oxygen uptake [13–15]), alternated by periods of active or passive rest. Wisløff *et al*. suggested a superior effect of HIT compared to continuous training (CT) on physical fitness, cardiovascular function and quality of life in HF patients [16]. This study applied an intensive HIT training regimen in HF patients and found large improvements in fitness levels. After this promising study, Fu *et al*. found similar results [17], but not all subsequent studies [18–22], reinforced the superior effect of HIT compared to CT in HF patients.

Previous studies that revealed a superior effect of HIT adopted relatively long bouts of high-intensity exercise (3–4 min) [16,17], followed by active ‘rest’ periods of 3 minutes at an intensity up to 70% of maximal heart rate. Performance of such long bouts of exercise at high-intensity and/or ‘rest’ periods at such vigorous intensity levels may not be feasible for all HF patients. Furthermore, most previous training studies have applied a training frequency of three times per week, which is time consuming. Whilst training frequencies of twice per week can be sufficient to induce a positive effect on fitness levels [23], no previous study examined the efficacy of HIT with lower training frequency or whether there is a difference between such HIT and CT training protocols. Therefore, we explored the benefits of a feasible HIT-protocol with high-intensity bouts of moderate duration (i.e. 1 min), the active rest at relatively low intensity (i.e. 30% maximal workload), and a training frequency of twice a week.

In this study, we examined whether 12-weeks of CT or HIT is effective and feasible for HF patients, and whether this HIT-protocol leads to superior effects on fitness, cardiovascular function and quality of life compared to CT. We hypothesize that the HIT-protocol is feasible, whilst the effects on physical fitness, cardiovascular function and quality of life in HF patients New York Heart Association (NYHA) class II-III are superior compared to CT.

## Methods

### Subjects

We included 29 patients (65±8 yrs) diagnosed with HF classified as NYHA class II-III, with a history of left ventricular ejection fraction (LVEF) ≤45% (assessed by 2D/4D echocardiography). Patients were recruited from the department of Cardiology of the Radboud university medical center and the Canisius-Wilhelmina Hospital (Nijmegen, the Netherlands) and through advertisements. Patients with HF due to congenital heart disease or HF caused by valve pathology were excluded. Other exclusion criteria were: diabetes mellitus (type 1 or 2), hypercholesterolemia (total cholesterol >6.5mmol/L), severe renal failure (glomerular filtration rate<30 mL/min/1.73m^2^), exercise-induced ischemia (i.e. ECG abnormalities suggestive for ischemia on maximal exercise testing), severe co-morbidities (e.g. COPD GOLD ≥3), pathology that restricts patients from participation to exercise (e.g. orthopedic/neurological disorders interfering with movement), pre-menopausal women or women on hormone replacement therapy, and subjects with contra-indications for maximal exercise testing [24]. Subjects had to be in a clinically and pharmacologically stable situation (>3 months) prior to participation. The Medical Ethical Committee of the Radboud university medical center approved this study (CMO region Arnhem-Nijmegen) on October 26^th^ 2010. This trial is registered in the Dutch Trial Register (NTR3671) 3 months after the start due to practical reasons. The authors confirm that all ongoing and related trials for this intervention are registered. Written informed consent was obtained from each subject before participation in this study. Subject recruitment was done between July 2011 and September 2014. Follow-up lasted until February 2015. This study was monitored by a data safety monitoring board, which also approved the submission of this study.

### Experimental protocol

Subjects reported to our laboratory for a medical screening, after which they were allocated to 12-weeks moderate-intensity CT or HIT. In addition, to assess changes over time in HF patients, we included a control group consisting of HF patients who were unable to participate in the training program due to geographical reasons or time-constraints, which was tested before and after a 12-week control period. Before and after the 12-week intervention/control period, subjects underwent a maximal incremental cycling test to determine physical fitness, echocardiography to examine cardiac function and structure, and vascular ultrasound measurements to examine peripheral artery vascular function and structure. Finally, questionnaires were used before and after the intervention to assess health-related quality of life and HF symptoms. All measurements were performed in the Radboud university medical center (Nijmegen, the Netherlands). Due to the nature of the study design and practical reasons, blinding participants and researchers was not possible.

### Measurements

#### Subject characteristics

We determined height, weight (Seca 888 Scale, Seca, Hamburg, Germany), body mass index, body fat percentage [25], and waist and hip circumference. Furthermore, we obtained heart rate and blood pressure (manually, WelchAllyn, Maxi-Stabil 3, NY, USA), an electrocardiogram to determine heart rhythm, and a venous blood sample to determine fasted glucose and (total) cholesterol concentrations.

#### Primary outcome—Physical fitness

An incremental maximal cycling test was performed on a cycle ergometer (Ergoline, Ergoselect 200k, Bitz, Germany). Subjects were instructed to pedal (>60rpm) whilst workload was increased 10–15 Watt/min, depending on the expected physical fitness of the participant (based on sex, age, height, and previous results on exercise testing). During exercise, breath-by-breath gas analysis was recorded continuously (LabManager V5.32.0). For the termination of maximal exercise testing we adhered to recent guidelines [24]. Peak oxygen uptake (VO_2peak_) was defined as the highest oxygen uptake (30-second average).

#### Secondary outcome—Physical fitness

Oxygen uptake at the anaerobic threshold (AT) was determined using the V-slope method [26]. Peak oxygen uptake and oxygen uptake at the AT was also expressed as a percentage of the predicted maximal oxygen consumption [27]. Ventilatory efficiency was defined as the slope of the ventilation to the carbon dioxide production (VE/VCO_2_ slope) calculated over the linear phase of the response up to the AT. The maximal oxygen pulse (oxygen consumption per heart rate, O_2_/HR) was determined (10-second average). The presence of chronotropic incompetence, defined as a heart rate reserve <80% of predicted [28], was noted. Prior to testing, all medication was continued.

#### Secondary outcome—Vascular function and structure

Subjects were prepared according to guidelines for the assessment of flow-mediated dilation (FMD) [29]. Subjects were instructed to continue medication, but to refrain from diuretics the day of testing for practical reasons. The measurements were performed in a temperature-controlled room (22.5±0.7°C). Prior to testing, subjects rested in the supine position for 10 minutes. Vascular function measurements were performed using a 10-MHz multifrequency linear array probe attached to a high-resolution ultrasound machine (Terason T3000, Burlington, MA, USA). We examined brachial and superficial femoral artery endothelial function using the FMD according to the guidelines [29]. Subsequently, we measured the brachial artery maximal diameter and blood flow responses to ischemic handgrip exercise, as described in previous studies [30,31]. The peak blood flow provides a valid and accepted index of resistance artery size and remodelling and the brachial artery diameter response for maximal dilating capacity [30]. We examined carotid artery intima-media thickness (IMT), which represents a surrogate measure for atherosclerosis [32]. Finally, we examined the endothelium-independent dilation of the brachial artery by examining the diameter response to an exogenous nitric oxide donor (sublingual administration of 400μg glyceryl trinitrate (GTN)).

#### Secondary outcome—Cardiac function

Transthoracic (4D) echocardiography was performed with an ultrasound scanner (Vivid E9, General Electric Healthcare, Horten, Norway) with M5-S and V4 probe according to the guidelines of the American Society of Echocardiography [33]. Echocardiographic images were analyzed post-hoc with EchoPAC software (version 112, General Electric Healthcare, Horten, Norway). From 4D-images we assessed: left ventricular end-diastolic volume, left ventricular end-systolic volume, stroke volume, LVEF, cardiac output, and cardiac index. Left ventricular longitudinal, circumferential, radial, and area strain were analyzed by 4D speckle tracking. Moreover, we measured the isovolumetric contraction and relaxation time from tissue Doppler tracings of the lateral and septal mitral annulus. To describe diastolic function we obtained the following parameters by (tissue) pulsed-wave Doppler tracings: peak mitral flow velocity during early (E) and late (A) diastole, the systolic (S) and diastolic (D) inflow velocity over the pulmonary valve, and the peak mitral annulus velocity during early filling (E’) of the lateral and septal mitral annulus. The E/A-ratio, S/D-ratio, and E/E’-ratio were calculated.

#### Secondary outcome–Questionnaires

To measure health-related quality of life, the SF-36 Health Survey was used [34,35]. Additionally, we used the Minnesota living with Heart Failure Questionnaire (MLHFQ), to measure patient perceptions of the effects of HF on their physical, psychological and socioeconomic lives [36].

### 12-week intervention

Training was performed twice a week for 12 weeks in a rehabilitation setting or hospital and was supervised by physiotherapists. When a participant missed a training session, this session was rescheduled to ensure a total of 24 training sessions (i.e. 100% compliance). Training was performed on a cycle ergometer (Lode Corival, Procare, Groningen, The Netherlands). Both the CT- and HIT-session started with a warm-up of 10-minutes at 40% of maximal workload (Watt) as obtained from the cardiopulmonary maximal exercise test at baseline, and concluded with a cooling-down of 5-minutes at 30% of maximal workload. Workload was increased during the 12-week training period based on the Borg scores of perceived exertion, to maintain a sufficient training stimulus when physical fitness was expected to improve.

#### CT-group

CT consisted of 30-minutes at 60–75% of maximal workload. Training intensity was controlled using the Borg score (scale 6–20) [37], aiming at a Borg score of 12–14 during the training session, as recommended in the current exercise guidelines for HF patients [12]. Borg score and heart rate were determined after the warm-up, at 20, 30 and 40 minutes of exercise, and after the cooling-down.

#### HIT-group

HIT consisted of 10 periods of 3.5-minutes of exercise, consisting of intervals of 1-minute at 90% of maximal workload, and 2.5-minutes at 30% of maximal workload, aiming at a Borg score of 15–17 during the high-intensity intervals. Borg score and heart rate were determined at the end of the warm-up, after repetition 1, 3, 7 and 10, and after the cooling-down.

#### Control group

Control subjects were instructed not to alter their daily physical activities.

### Statistical analysis

We have made a pre-study sample size calculation based on previous studies examining the difference in effect between CT and HIT. Some studies suggest n = 2–3 per group is sufficient [16,17], whilst data from others suggest several thousand subjects must be recruited to detect differences between CT and HIT [19]. We rationalized that n = 10–20 will provide (clinically) meaningful insight into the effect of CT *versus* HIT. Therefore, using a conservative approach (accounting for drop-outs), we aimed for n = 20 for both exercise training groups (and n = 10 in the control group). Data was analyzed using IBM SPSS Statistics 20.0 (IBM Corp., Armonk, NY, USA). Parameters were checked for normality using a Kolmogorov-Smirnov test. When data was not normally distributed, a non-parametric alternative was used or natural logarithmic data transformation was applied. Categorical and nominal parameters were compared with a Chi-Square test. Baseline characteristics of the groups were compared with a 1-way ANOVA or Kruskal-Wallis test when data was not normally distributed. A 2-way repeated measures ANOVA was used to examine the impact of exercise training (time-effect), and whether the change differs between HIT and CT (time*group-effect). When a significant main effect (time) or interaction-effect (time*group) was observed, post-hoc tests with least-significant difference were used to identify differences between and within groups. When data for this 2-way comparison was not normally distributed, we used individual tests to examine the effect of time, group and time*group. Changes in the control group were tested with a paired Student’s *t*-test, or Wilcoxon test when data was not normally distributed. To control for the potential impact of baseline diameter on FMD [38], we used logarithmically transformed diameter data and adopted a univariate General Linear Model with baseline arterial diameter as a covariate, to compare differences between groups. Potential drop-outs were left out of the analysis and were not replaced. A Pearson correlation coefficient was determined for the relation between baseline quality of life and exercise-induced changes in quality of life. Data are presented as mean±standard deviation (SD), unless stated otherwise. Significance level was set at P<0.05.

## Results

**Fig 1:**
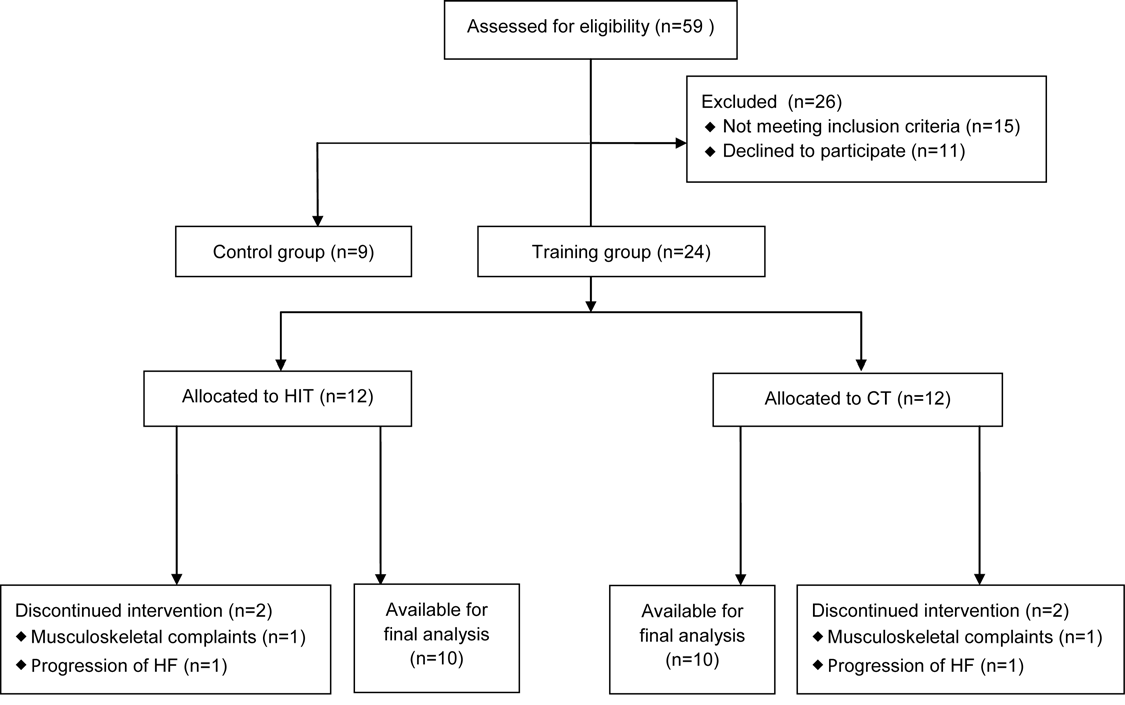
Flow-chart of the inclusion of subjects.

For this study, 59 HF patients were screened. Fifteen patients did not meet inclusion criteria (screen failures) and 11 patients declined to participate after screening due to lack of time (n = 10) or change in health (n = 1) (Fig 1). We allocated 24 patients to HIT or CT. Nine HF patients were included in the control group (non-randomized). In both the HIT- and CT-group, 2 drop-outs were reported after allocation (71±2yrs; male:female 3:1; NYHA class II:III 3:1), which makes the total drop-out 17%. A patient in both training groups dropped out due to musculoskeletal complaints and a patient in both training groups dropped out due to progression of HF. Twenty patients in the training-groups and nine controls completed the study and were available for final analysis. The groups were not significantly different in age, body mass index, NYHA class, etiology of HF, blood pressure, heart rate, LVEF and physical fitness (Table 1). The control group consisted of significantly more females than the CT-group (P = 0.028, Table 1). Cardiovascular medication use is documented in Table 1.

**Table 1:** Subject characteristics and cardiovascular medication.

	CT (n = 10)	HIT (n = 10)	Control (n = 9)	
Age (yrs)	64±8	63±8	67±7	0.57
Sex (male:female)	10:0*	9:1	5:4	0.028
Body weight (kg)	89.7±11.9	87.6±23.6	77.0±10.5	0.16
Height (cm)	177±5	177±3	174±9	0.66
Body mass index (kg/m^2^)	28.9±4.7	28.1±7.5	25.4±2.7	0.24
NYHA class (II:III)	8:2	8:2	8:1	0.84
Etiology (Isch:Non-isch)	8:2	7:3	5:4	0.51
Systolic blood pressure (mmHg)	132±23	132±18	130±25	0.98
Diastolic blood pressure (mmHg)	83±11	79±10	78±14	0.48
Resting heart rate (/min)	59±11	57±7	60±10	0.80
Maximal heart rate (/min)^†^	129±19	126±16	120±15	0.53
Chronotropic incompetence (yes:no)^†^	5:4	8:2	6:1	0.33
VO_2peak_ ^†^ (mL/min/kg)	21.0±3.4	19.1±4.1	17.4±5.8	0.26
VO_2peak_ ^†^ (% of predicted VO_2peak_)	86±8	79±17	81±22	0.63
LVEF (%)	38±6	37±6	40±11	0.84
Medication				
Angiotensin converting enzyme-inhibitors	5 (50%)	6 (60%)	8 (89%)	0.19
Angiotensin II receptor antagonists	4 (40%)	4 (40%)	1 (11%)	0.30
Aldosterone antagonist	6 (60%)	7 (70%)	8 (89%)	0.36
Diuretics (loopdiuretics)	7 (70%)	6 (60%)	4 (44%)	0.50
β-blockers	10 (100%)	9 (90%)	9 (100%)	0.37
Antiplatelet drugs	6 (60%)	4 (40%)	3 (33%)	0.47
Coumarin derivates	4 (40%)	7 (70%)	4 (44%)	0.35
Statins	10 (100%)	9 (90%)	4 (44%)^§^	0.007

Data is presented as mean±SD. P-values refer to a 1-way ANOVA.

^†^ Data was unavailable for 1 subject in the CT-group and 3 subjects in the control-group.

* Significantly less females compared to the control-group.

^§^Lower compared to CT-group and HIT-group.

### Exercise training

When averaging all training sessions, CT was performed at 66±5% of maximal workload, whilst the high-intensity intervals during HIT were performed at 102±7% of maximal workload (P<0.001). CT was performed at 81±7% of maximal heart rate, and the high-intensity intervals during HIT were performed at 83±9% of maximal heart rate (P = 0.70). Borg-scores during CT and HIT were 13±1 and 14±1 respectively (P = 0.27).

### Impact of exercise training

#### Physical fitness

VO_2peak_ tended to increase after training (P = 0.06), whilst VO_2peak_/kg did not change after exercise (P = 0.10). A significant increase after training was found in the VO_2peak_ related to predicted VO_2peak_ (%), maximal workload, oxygen uptake at the AT and maximal oxygen pulse (Table 2). No significant differences were observed between both interventions (Table 2).

**Table 2:** Maximal incremental cycling test.

	CT (n = 10)			HIT (n = 10)		P-value		
	*Pre*	*Post*	*Pre*		*Post*	*Time*	*Group*	Time*Group
VO_2peak_ (mL/min)	1881±214	1887±27	1662±562		1792±559	0.06	0.44	0.08
VO_2peak_ (mL/min/kg)	21.2±3.6	21.3±3.7	19.1±4.1		20.4±4.3	0.10	0.14	0.09^#^
VO_2peak_ (% pred. VO_2peak_)	86±8	87±10	79±17		85±16	0.044	0.48	0.08
Max. workload (Watt)	145±22	152±26	126±38		142±45	<0.001	0.24	0.07
Max. heart rate (/min)	129±19	132±24	126±16		125±15	0.78	0.30	0.51
VE/VCO_2_ slope	32.2±3.3	32.7±5.8	28.7±5.8		29.4±7.7	0.52	0.18	0.91
VO_2_ at AT (mL)	1030±287	1248±388	1033±319		1090±225	0.041	0.54	0.22
Max. O_2_/HR (mL)	16.2±2.2	16.7±2.8	14.0±4.0		15.4±3.8	0.006	0.25	0.13

Data is presented as mean±SD. P-values refer to a 2-way repeated measures ANOVA between the two training groups. One subject in the CT-group did not reach VO_2peak_, and therefore only VE/VCO_2_ slope and VO_2_ at AT could be determined.

^#^ For statistical reasons, data was analyzed with three separate tests to determine *time*, *group* and time*group P-values.

#### Vascular function/structure

We found no significant changes in brachial and superficial femoral artery diameter, peak blood flow, and FMD (Table 3). No change in endothelium-independent dilation of the brachial artery was observed after training for both groups (Table 3). Furthermore, we found no significant impact of HIT or CT on carotid artery IMT or IMT-to-lumen ratio (Table 3).

**Table 3:** Brachial (BA) and superficial femoral artery (SFA) endothelium-dependent vasodilation through flow-mediated dilation (FMD), peak diameter and endothelium-independent dilation (GTN), and common carotid artery (CCA) intima-media thickness (IMT).

	CT (n = 10)		HIT (n = 10)		P-value		
	*Pre*	*Post*	*Pre*	*Post*	*Time*	*Group*	Time*Group
BA diameter (mm)	4.5±0.5	4.5±0.5	4.4±0.9	4.4±0.8	0.83	0.68	0.86
BA FMD (%)	5.2±2.5	4.8±3.0	5.3±2.6	4.7±2.5	0.33	0.92	0.98
BA FMD (%, scaled)	5.3±2.5	4.8±2.5	5.2±2.5	4.6±2.5	0.47	>0.999	0.91^#^
BA SR_AUC_ (s, 10^3^)	19.9±9.6	18.6±7.4	17.8±9.2	22.3±7.7	0.42	0.80	0.14
BA GTN (%)	17.6±7.0	16.3±6.5	16.8±8.7	15.2±4.9	0.33	0.74	0.92
BA GTN (%, scaled)	17.9±4.5	16.6±4.5	16.1±4.5	14.6±4.5	0.42	0.27	0.94
BA FMD-GTN ratio	0.34±0.21	0.33±0.24	0.42±0.30	0.34±0.15	0.46	0.63	0.82
BA CADC (%)	10.6±6.4	11.3±4.8	16.1±7.4	13.4±5.5	0.52	0.12	0.26
BA CADC (%, scaled)	11.0±4.2	12.0±4.2	15.0±4.2	12.8±4.2	0.72	0.11	0.27
BA peak blood flow (mL/min)	794±139	862±261	711±264	556±165	0.38	0.56	0.46
SFA diameter (mm)	7.1±1.2	6.9±1.2	6.7±1.1	6.5±1.0	0.38	0.48	0.88
SFA FMD (%)	3.3±1.9	5.1±4.8	4.2±1.8	3.4±2.8	0.58	0.74	0.19
SFA FMD (%, scaled)	3.5±2.9	5.1±2.9	4.1±2.9	3.1±2.9	0.74	0.50	0.19
SFA SR_AUC_ (s, 10^3^)	9.2±5.6	10.4±10.8	13.5±7.2	6.9±3.9	0.11	0.80	0.07
CA IMT (mm)	0.80±0.13	0.76±0.20	0.72±0.12	0.67±0.16	0.20	0.24	0.96
CA IMT-to-lumen ratio	0.12±0.02	0.11±0.02	0.11±0.02	0.11±0.02	0.40	0.59	0.45

Data is presented as mean±SD. P-values refer to 2-way repeated measures ANOVA between the 2 training groups.

^#^ For statistical reasons, data was analyzed with three separate tests to determine *time*, *group* and time*group P-values. Due to technical problems, BA GTN/FMD-GTN ratio/peak blood flow was available for 9 subjects in the HIT-group and SFA FMD was available for 8 subjects in the HIT-group. CCA IMT and IMT-to-lumen ratio were available for 8 subjects in each group. SR_AUC_; shear rate area-under-the-curve. CADC; conduit artery dilating capacity.

#### Cardiac function/structure

Most of the parameters of cardiac systolic function, left ventricle strain, or diastolic function demonstrated no change after HIT or CT (Table 4). Negligible but significant changes were found in area strain and isovolumetric contraction time (Table 4).

**Table 4:** Echocardiographic left ventricular volumes, systolic function, strain and diastolic function.

	CT (n = 10)		HIT (n = 10)		P-value		
Systolic function	*Pre*	*Post*	*Pre*	*Post*	*Time*	*Group*	Time*Group
LVEDV (ml)	154±24	159±28	194±39	204±44	0.26	0.002	0.64^#^
LVESV (ml)	98±14	102±19	134±32	132±40	0.87	0.037	0.63
Stroke volume (ml)	56±13	57±13	61±14	72±16	0.06	0.16	0.12
LVEF (%)	36±4	36±5	32±7	36±9	0.09	0.57	0.07
Cardiac output (L/min)	3.5±0.6	3.4±0.7	3.5±0.8	4.3±1.0	0.20	0.21	0.07
Cardiac index (L/min/m^2^)	1.7±0.3	1.6±0.3	1.7±0.4	2.1±0.5	0.22	0.14	0.08
Longitudinal strain (%)	-9±3	-9±3	-9±3	-8±3	0.60	0.47	0.38^#^
Circumferential strain (%)	-10±2	-10±3	-9±3	-8±3	0.22	0.43	0.19
Radial strain (%)	23±7	22±6	23±8	20±8	0.13	0.71	0.48
Area strain (%)	-17±4	-15±6	-17±5	-14±5	0.044	0.73	0.97
IVCT-L (ms)	52±7	50±10	49±12	58±7	0.18	0.56	0.029
IVCT-S (ms)	57±14	59±11	53±9	56±11	0.35	0.46	0.87
**Diastolic function**							
IVRT-L (ms)	145±32	149±27	142±27	159±27	0.13	0.75	0.38
IVRT-S (ms)	160±36	148±22	164±41	170±37	0.60	0.45	0.22^#^
E/A	1.15±0.71	1.17±0.89	1.53±1.42	1.60±1.53	0.49	0.36	0.59^#^
S/D	1.38±0.74	1.17±0.34	1.00±0.40	1.26±0.59	0.85	0.48	0.14
E/E’-L	6.8±1.9	6.7±1.9	10.3±4.4	9.8±6.3	0.71	0.07	0.74
E/E’-S	10.1±4.1	11.1±5.2	12.6±9.8	11.8±11.2	0.93	0.67	0.42

Data is presented as mean±SD. P-values refers to 2-way repeated measures ANOVA between the 2 training groups.

^#^ For statistical reasons, data was analyzed with three separate tests were performed to determine *time*, *group* and time*group P-values. 4D data was available for 7 patients in the CT-group and 8 patients in the HIT-group. IVCT-l, IVRT-C, IVRT-S and E/E’-L was available for 9 patients in the HIT-group. IVCT-S and E/E’-S was available for 8 patients in the HIT-group. IVCT-L and S/D ratio was available for 9 subjects in the CT-group. IVRT-L and E/A ratio was available for 8 subjects in the CT-group.

LVEDV; left ventricular end-diastolic volume. LVESV; left-ventricular end-systolic volume. IVCT-L/S: isovolumetric contraction time, lateral/septal. IVRT-L/S; isovolumetric relaxation time, lateral/septal. E/A ratio; peak mitral flow velocity during early filling/peak mitral flow velocity during atrial contraction. S/D; systolic flow velocity pulmonary vein/diastolic flow velocity pulmonary vein. E/E’-L/S; peak mitral flow velocity during early filling/peak mitral annulus velocity during early filling, lateral/septal.

#### Quality of life

There was no significant change in the SF-36 total score (Table 5). There was a significant increase in the SF-36 subscale 'physical function' after training (P = 0.004, Table 5), which did not differ between groups (time*group P = 0.11). A trend for an inverse correlation was found between baseline SF-36 scores and training-induced change in SF-36 scores (r = -0.51, P = 0.052). We found no change in the MLHFQ for both groups (Table 5). No significant correlations were found between baseline MLHFQ scores and training-induced change in MLHFQ.

**Table 5:** Results of the SF-36 and Minnesota living with HF questionnaire (MLHFQ).

	CT (n = 9)		HIT (n = 8)		P-value		
**SF-36**	*Pre*	*Post*	*Pre*	*Post*	*Time*	*Group*	Time*Group
Physical functioning	74±22	78±17	57±21	69±17	0.004	0.16	0.11
Physical health subscore^†^	73±20	76±16	60±22	67±19	0.15	0.26	0.52
Mental health subscore^†^ ^,^ ^‡^	81±9	83±10	83±5	82±10	0.75	0.87	0.54
Total score^†^ ^,^ ^‡^	75±16	78±13	68±14	73±14	0.18	0.42	0.76
	**CT (n = 9)**		**HIT (n = 10)**		**P-value**		
**MLHFQ**	*Pre*	*Post*	*Pre*	*Post*	*Time*	*Group*	Time*Group
Total score	18±14	16±16	21±15	20±14	0.81	0.56	0.89

Data is presented as mean±SD. P-values refers to 2-way repeated measures ANOVA between the 2 training groups. Results of the SF-36 were scored on a 0–100 scale, in which a high score represents a better quality of life. Results of the MLHFQ were scored on a 0–105 scale, in which a low score indicates few HF-related complaints.

^†^ Data was available for 7 subjects in the HIT-group.

^‡^ Data was available for 8 subjects in the CT-group.

#### Control group

We found no changes over the 12-week period in maximal oxygen uptake (17.4±5.9 *versus* 17.5±5.8 ml/min/kg, P = 0.79) or in any of the other parameters of physical fitness (all P>0.05, S1 Table). Except for a decrease across time in the superficial femoral artery FMD and an increase in lateral E-E’-ratio, we found no changes in cardiac and vascular structure or function or in the SF-36 score and MLHFQ in controls (all P>0.05, S1 Table).

## Discussion

This study comprehensively compared physical fitness, vascular function, cardiac function and quality of life between a feasible and practical HIT-protocol *versus* traditional CT in HF patients. We have demonstrated that CT and HIT are both feasible in HF patients and induced a significant improvement in measures of (sub)maximal exercise performance and fitness, with no significant differences between both types of exercise training. Despite these changes in fitness, we did not find improvement in measures of resting cardiac and vascular structure and function. Furthermore, except an improvement in the subscale ‘physical function’ that may be related to the change in fitness, no effect of training was found on quality of life. Therefore, our data suggest that both types of exercise training successfully improve measures of physical fitness within 12-weeks, but not cardiovascular function at rest or quality of life. Moreover, we found no evidence for superiority of this more feasible HIT-protocol over traditional CT on the parameters presented in our study.

### Physical fitness

Previous work found improvements in physical fitness in HF after HIT ranging from 8–46% [16,20,21], whilst studies adopting CT found changes ranging between 0–22% [17,19]. Most of these training studies have included low numbers of participants, which may explain the large variation in results. The largest HF exercise training study so far (>1,000 participants), the HF-ACTION trial, reported a median increase in fitness of 4% [39]. Although in the lower ends of the spectrum, the change in physical fitness in our study (~4%) is within the range of improvements as reported in previous work. As demonstrated in various previous studies, training characteristics (e.g. frequency, intensity and duration) are important factors determining training responses [40]. Nonetheless, our relatively low-frequency protocols were sufficient to induce significant improvements in fitness levels. Similar observations were made by Belardinelli *et al* [23], who adopted a long-term (10-year), low-frequency exercise training program in HF patients. They reported improvement in physical fitness levels in trained subjects after 1 year of training, whilst fitness levels remained higher than in controls across the 10-year intervention. In addition to the low frequency of training, the relatively modest changes in fitness in our study may relate to characteristics of the included participants, such as genetic factors [41] or to *a priori* higher levels of physical fitness as lower physical fitness levels are associated with larger training-induced improvements in HF patients [42,43]. Indeed, some previous HIT-studies demonstrated large improvement after exercise training in HF patients with low baseline levels of physical fitness [16–18]. Moreover, patients with chronotropic incompetence (i.e. 68% in our study) have attenuated exercise-induced improvement in parameters of fitness, which could have affected the effect size in our study [44]. Despite the relatively modest effect sizes, our exercise training protocols were successful in improving parameters of physical fitness.

We did not demonstrate significant differences between CT and HIT on the change in physical fitness, a finding which is in agreement with some [18,19,22], but not all previous studies [16,17]. Interestingly, recent meta-analyses suggest that (high-intensity) interval training results in superior effects on physical fitness compared to CT in HF [45,46]. Despite these promising results of HIT in HF, larger trials that focus on clinical end-points are needed [47]. Also, limitations of a meta-analysis should be taken into consideration, as both HIT and CT interventions included in these meta-analyses comprise of many different protocols and exercise intensities. Especially for HIT, it is suggested that the time spent at a high percentage of peak oxygen uptake, determined by intensity and duration of the work and rest intervals, importantly contributes to the effect size [48]. Whilst the intensity of our high-intensity-bouts is high, the time spent at these high-intensity bouts was lower than in previous studies demonstrating superior effects of HIT compared to CT [16,17]. Furthermore, to validly compare exercise training regimes, it is important that total workload is not significantly different. A study that compared training-effects of HIT and CT and applied individually designed training programs with specific emphasis on comparable workloads for HIT and CT, found no differences between training modalities [19]. Therefore, more rigorous exercise programs (both in frequency, duration, and training load) rather than the type of training *per se* may contribute to larger improvements in physical fitness.

### Cardiac and vascular adaptation

Exercise in HF patients is associated with beneficial cardiac remodelling [8]. After 12-weeks of training, we found no improvements in cardiac structure and function at rest, although the increase in maximal oxygen pulse suggests an increase in stroke volume during exercise. Previous studies that reported significant changes in cardiac function or structure generally applied training periods ≥6 months [8]. Therefore, the relatively short duration of training may contribute, at least partly, to the lack of cardiac remodelling in our study. Moreover, the largest proportion of our training-participants reported ischemic HF etiology. This could be of special importance, since a recent study suggested that cardiac adaptation during outpatient rehabilitation is more prominent in HF patients with non-ischemic etiology [49]. Furthermore, our results are in line with a recent meta-analysis that could not confirm that HIT is superior to CT to induce cardiac adaptations [46].

In our study, we found no superior effect of HIT to improve vascular function, which contrasts with the findings of a recent meta-analysis [50]. However, 4 out the 7 studies included in this meta-analysis showed a superior effect of HIT. Interestingly, these studies were all from the same laboratory and did not follow contemporary guidelines to assess endothelial function. We found no overall effect of exercise training on vascular function. As previous literature has reported a wide range of exercise-induced responses (and also demonstrated non-responders) in vascular function, Green *et al*. investigated which factors predict this response [51]. They concluded that exercise-induced improvements in vascular function are associated with a lower pre-training vascular function. When comparing the baseline FMD values of our subjects to normal values published previously [52], the endothelial function of the subjects in our study was within the normal range for their age. This may relate to the optimal pharmacological therapy of the HF patients we included [53]. Moreover, we have previously demonstrated that the shear rate pattern during exercise (i.e. an important stimulus for exercise-induced adaptation in vascular function) is less beneficial in HF patients compared to controls [54]. Preserved FMD before training and a suboptimal shear rate stimulus during exercise training may contribute to the absence of a significant training-induced change in vascular function.

### Quality of life

Quality of life after exercise training in HF patients is previously demonstrated to improve [7,55]. In our study, we indeed found improvement in the subscale for physical functioning after exercise training, but not for total quality of life. The lack of improvement in quality of life may relate to the relatively ‘good’ quality of life at baseline, which was well above that of previous studies [17,56,57] and consequently, provides little space for further improvement. In support of this idea, we observed a trend for an inverse relation between baseline SF-36 and change in SF-36 in the training group. This indicates that subjects with lower quality of life prior to exercise training demonstrate a larger benefit from the intervention. Furthermore, the inclusion of more relatively old HF patients in our study may also contribute to the smaller effect size of exercise training on quality of life, as demonstrated in a previous study [58]. This latter study found that older HF patients (>60 yrs) demonstrate a smaller effect of exercise training on quality of life compared to younger HF patients (<60 yrs).

### Clinical relevance

Given the importance of (even small improvements in) fitness levels for the prognosis of HF patients [5,6,59], finding both a feasible and effective training program is clinically relevant. Another important factor is adherence to exercise training, which often is reported to be low in HF patients due to time-constraints and lack of energy [60]. A HIT training program with lower training frequency and high-intensity intervals of moderate duration might address these two major factors of non-compliance. Therefore, we have studied whether such a program is effective and whether it is superior to CT. The results of this study suggest that low frequency HIT with high-intensity intervals of moderate duration is feasible and successful in improving fitness. Such findings may be of clinical relevance and future studies should therefore focus on finding the optimal exercise protocol for HF patients to achieve long-term benefits and adherence.

### Limitations

Although we have included a relatively small number of patients, our sample size was in line with previous studies that demonstrated differences in effects of HIT and CT on physical fitness [16,17] and vascular function [50]. Moreover, we have used state-of-the-art techniques for measuring physical fitness and vascular function, in contrast to some previous studies that used suboptimal techniques to assess endothelial function. Therefore, we *a priori* expected to have sufficient power to detect significant differences between HIT and CT. Post-hoc power analysis revealed a power of 54% to detect differences in change in physical fitness between the two types of training. Finally, we did not provide a comparison between HIT with short high-intensity bouts and HIT with long high-intensity bouts. We encourage future studies to investigate whether different HIT-protocols render different results and to elucidate the optimal HIT-protocol.

In conclusion, we have demonstrated that 12-weeks of exercise training in HF patients is associated with improvements in parameters of physical fitness, whilst no improvements in cardiovascular function at rest or (total) quality of life are observed. Moreover, our data does not provide strong evidence for a potentially superior improvement in physical fitness, cardiovascular function or quality of life after 12-weeks of HIT compared to CT in HF patients NYHA class II-III.

## Supporting Information

S1 TREND Checklist TREND statement checklist. (PDF)

S1 Protocol Research protocol. (PDF)

S1 Table Results of the control group. Data is presented as mean ±SD. ^†^Data missing for 1 control subject. VO_2peak_; peak oxygen uptake. AT; anaerobic threshold. BA; brachial artery. SFA; superficial femoral artery. GTN; glyceryl trinitrate. CADC; conduit artery dilating capacity. IMT; intima-media thickness. SR_AUC_; shear rate area-under-the-curve. CADC; conduit artery dilating capacity. LVEDV; left ventricular end-diastolic volume. LVESV; left-ventricular end-systolic volume. IVCT-L/S: isovolumetric contraction time, lateral/septal. IVRT-L/S; isovolumetric relaxation time, lateral/septal. E/A ratio; peak mitral flow velocity during early filling/peak mitral flow velocity during atrial contraction. S/D; systolic flow velocity pulmonary vein/diastolic flow velocity pulmonary vein. E/E’-L/S; peak mitral flow velocity during early filling/peak mitral annulus velocity during early filling, lateral/septal. MLHFQ; Minnesota living with heart failure questionnaire.
(DOCX)
