# Supplementary material for: Effects of High-Intensity Interval Training versus Continuous Training on Physical Fitness, Cardiovascular Function and Quality of Life in Heart Failure Patients
Source: PLoS One. 2015 Oct 30;10(10):e0141256. doi: 10.1371/journal.pone.0141256 (PMC4627811; doi:10.1371/journal.pone.0141256)
Supplement: S1 Protocol — (PDF) [file pone.0141256.s002.pdf]

ONDERZOEKS PROTOCOL

(Mei 2014)

PROTOCOL TITLE ‘*Train hard is smart for the heart; an exercise study on clinical outcome and mechanisms in heart failure patients*’

**Protocol ID**	
**Short title**	**Train hard is smart for the heart**
**Version**	6
**Date**	09-05-2014
**Coordinating investigator/project leader**	**Prof. Dr. Maria T.E. Hopman**<br>e-mail: maria.hopman@radboudumc.nl<br>telefoon: 024-3614200
**Principal investigator(s) (in Dutch: hoofdonderzoeker/uitvoerder)**<br>***Multicenter research: per site***	**Dr. Dick H.J. Thijssen**<br>e-mail: dick.thijssen@radboudumcn.nl<br>telefoon: 024-3614222
**Sponsor (in Dutch: verrichter/opdrachtgever)**	**Afdeling Fysiologie, UMC St Radboud**<br>Telefoon: 024-3613650
**Independent physician(s)**	**Prof. Dr. Cees Tack**
**Laboratory sites <*if applicable*>**	**Niet van toepassing**
**Pharmacy <*if applicable*>**	**Niet van toepassing**

PROTOCOL SIGNATURE SHEET

Name	Signature	Date
**Sponsor or legal representative:**<br><br>**For non-commercial research,**<br>**Head of Department:**<br>***Prof. Dr René Bindels***		**8 april 2010**
**Coordinating Investigator/Project leader/Principal Investigator:**<br>***Dr Dick H.J. Thijssen***		**8 april 2010**

TABLE OF CONTENTS

1. INTRODUCTION AND RATIONALE 9
2. OBJECTIVES 9
3. STUDY DESIGN 11
4. STUDY POPULATION 14
4.1 Population (base) 14
4.2 Inclusion criteria 14
4.3 Exclusion criteria 14
4.4 Sample size calculation 14
5. TREATMENT OF SUBJECTS 15
5.1 Investigational product/treatment 15
5.2 Use of co-intervention (if applicable) 15
5.3 Escape medication (if applicable) 15
6. INVESTIGATIONAL MEDICINAL PRODUCT 16
6.1 Name and description of investigational medicinal product 16
6.2 Summary of findings from non-clinical studies 16
6.3 Summary of findings from clinical studies 16
6.4 Summary of known and potential risks and benefits 16
6.5 Description and justification of route of administration and dosage 16
6.6 Dosages, dosage modifications and method of administration 16
6.7 Preparation and labelling of Investigational Medicinal Product 16
6.8 Drug accountability 16
7. METHODS 17
7.1 Study parameters/endpoints 17
7.1.1 Main study parameter/endpoint 17
7.1.2 Secondary study parameters/endpoints (if applicable) 17
7.1.3 Other study parameters (if applicable) 17
7.2 Randomisation, blinding and treatment allocation 17
7.3 Study procedures 17
7.4 Withdrawal of individual subjects 19
7.4.1 Specific criteria for withdrawal (if applicable) 19
7.5 Replacement of individual subjects after withdrawal 19
7.6 Follow-up of subjects withdrawn from treatment 19
7.7 Premature termination of the study 20
8. SAFETY REPORTING 21
8.1 Section 10 WMO event 21
8.2 Adverse and serious adverse events 21
8.2.1 Suspected unexpected serious adverse reactions (SUSAR) 21
8.2.2 Annual safety report 22
8.3 Follow-up of adverse events 23
8.4 Data Safety Monitoring Board (DSMB) 23
9. STATISTICAL ANALYSIS 24

9.1 Descriptive statistics 24
9.2 Univariate analysis 24
9.3 Multivariate analysis 24
9.4 Interim analysis (if applicable) 24
10. ETHICAL CONSIDERATIONS 25
10.1 Regulation statement 25
10.2 Recruitment and consent 25
10.3 Objection by minors or incapacitated subjects (if applicable) 25
10.4 Benefits and risks assessment, group relatedness 25
10.5 Compensation for injury 26
10.6 Incentives (if applicable) 27
11. ADMINISTRATIVE ASPECTS AND PUBLICATION 28
11.1 Handling and storage of data and documents 28
11.2 Amendments 28
11.3 Annual progress report 28
11.4 End of study report 29
11.5 Public disclosure and publication policy 29
12. REFERENCES 30

LIST OF ABBREVIATIONS AND RELEVANT DEFINITIONS

**ABR**	**ABR form (General Assessment and Registration form) is the application form that is required for submission to the accredited Ethics Committee (ABR = Algemene Beoordeling en Registratie)**
**AE**	**Adverse Event**
**AR**	**Adverse Reaction**
**CA**	**Competent Authority**
**CCMO**	**Central Committee on Research Involving Human Subjects**
**CV**	**Curriculum Vitae**
**DSMB**	**Data Safety Monitoring Board**
**EU**	**European Union**
**EudraCT**	**European drug regulatory affairs Clinical Trials GCP Good Clinical Practice**
**IB**	**Investigator's Brochure**
**IC**	**Informed Consent**
**IMP**	**Investigational Medicinal Product**
**IMPD**	**Investigational Medicinal Product Dossier**
**METC**	**Medical research ethics committee (MREC); in Dutch: medisch ethische toetsing commissie (METC)**
**(S)AE**	**Serious Adverse Event**
**SPC**	**Summary of Product Characteristics (in Dutch: officiële productinfomatie IB1-tekst)**
**Sponsor**	**The sponsor is the party that commissions the organisation or performance of the research, for example a pharmaceutical company, academic hospital, scientific organisation or investigator. A party that provides funding for a study but does not commission it is not regarded as the sponsor, but referred to as a subsidising party.**
**SUSAR**	**Suspected Unexpected Serious Adverse Reaction**
**Wbp**	**Personal Data Protection Act (in Dutch: Wet Bescherming Persoonsgevens)**
**WMO**	**Medical Research Involving Human Subjects Act (Wet Medisch-wetenschappelijk Onderzoek met Mensen**

SUMMARY

Rationale:

In Western countries, heart failure (HF) is a major cause of death. Despite current advances in the pharmacological management of HF, the prevalence is rapidly increasing and the prognosis remains poor. Physical fitness is the single best predictor of both cardiac and all-cause deaths among patients with cardiovascular disease and outperforms ejection fraction as a prognostic index (for survival) in HF. Despite the overwhelming evidence to promote physical activity, little is known regarding the type of exercise that yields optimal beneficial effects in HF. Some studies in healthy subjects or those with cardiovascular risk suggest greater fitness and cardiovascular adaptations after high intensity exercise than with 'traditional' moderate exercise. The rationale is that high intensity exercise (i.e. short bouts of exercise at ~90% of the maximal heart rate) allows patients to complete work at higher workload/intensity, but for a short period of time, inducing beneficial peripheral adaptations in vessels and muscles, without overloading the heart. A sound comparison between the effects of 'traditional' moderate *versus* high intensity exercise training in HF patients has never been examined.

Peripheral factors, such as endothelial dysfunction and increased vascular tone, are fundamental to the pathogenesis of HF. These peripheral vascular changes can be explained through changes in dilator (i.e. nitric oxide) *and* constricting (i.e. endothelin-1) pathways. However, the mechanisms responsible for the exercise-related improvement are not fully understood. Reversing the peripheral vascular changes most likely contributes to the positive effects of exercise in heart failure. While evidence supports a role for nitric oxide to partly explain the beneficial effects of 'traditional' exercise, no previous study examined the impact of high intensity exercise on nitric oxide in HF.

Objective: The overall aim of this project is to compare acute and chronic effects of moderate *versus* high intensity exercise training in HF patients. Specifically, we will:

1. *Compare the effects of 'traditional' moderate intensity versus novel high intensity exercise training in heart failure patients (NYHA-class II/III).* To this end, physical fitness, clinical outcome and cardiovascular function will be examined before and after 12-week of exercise training.
2. *Examine the impact of moderate as well as high intensity exercise training in heart failure patients (NYHA-class II/III) on the NO-pathway*. Therefore, I will examine the nitric oxide-mediated endothelial function before and after the 12-week interventions.

Study design: Randomised intervention study

Study population: 50 patients diagnosed with heart failure

Intervention (if applicable): Subjects will be randomly allocated to a 12-week intervention: 1. moderate-intensity exercise training, 2. high-intensity exercise training, or 3. control.

Main study parameters/endpoints:

1) Physical fitness (measured with a maximal cycling test)
2) NO-mediated endothelium-(in)dependent vasodilation of the forearm resistance arteries

Nature and extent of the burden and risks associated with participation, benefit and group relatedness:

Exercise training is not associated with a health risk. Moreover, exercise training typically causes a decreased cardiovascular risk, whilst vascular and cardiac function and structure improve after a period of exercise training. Also a number a previous studies have demonstrated that the cardiac workload during high intensity training is not significantly different to the (traditional)moderate-intensity training. Some studies have even demonstrated that the beneficial effects of exercise on remodelling of the heart are superior during high-intensity training compared with traditional moderate-intensity training in subjects with heart failure. Therefore, both types of exercise are not associated with an increased risk for development of health-related problems.

INTRODUCTION AND RATIONALE

Aging of the population and prolongation of the lives of cardiac patients has led to an increased prevalence of heart failure (HF). HF is a major cause of death,[1] exceeding mortality rates of most cancers. Annually, ~35000 new HF diagnoses are made, ~25000 patients are hospitalized and ~20% of HF patients die,[2] in the Netherlands.[3] Accurate therapeutic interventions for HF patients are mandatory.

Despite advances in the pharmacological and technological management, the prognosis of HF remains poor. Physical fitness outperforms ejection fraction as a prognostic index in HF.[4] Moreover, physical fitness is the single best predictor of both cardiac and all-cause deaths among patients with cardiovascular disease.[5] Therefore, exercise training may be efficient as a treatment for HF patients. Indeed, 'traditional' moderate intensity exercise increases fitness levels and improves survival and clinical outcome in HF.[6-8] Despite the overwhelming evidence for the benefits of exercise, no previous study has attempted to optimise exercise training or examined different types of exercise in HF.

Some recent data suggest greater fitness and cardiovascular adaptations after high intensity exercise than with 'traditional' moderate level exercise training in coronary artery disease[9], left ventricular dysfunction[10] and healthy subjects.[11] High intensity training is performed at ~90% of the maximal heart rate.[12] The rationale for high-intensity training is that it allows patients to complete work at higher intensity for a short period of time, inducing beneficial peripheral adaptations without overloading the heart.[13] Whilst HF patients are able to perform high intensity exercise bouts,[13, 14] it is unknown whether high intensity training has favorable effects over 'traditional' exercise training.

A recent pilot study, performed in a selective (n=9, 76 yrs, mainly men) group of severe HF patients, suggested that high intensity training may be more efficient than moderate intensity training to improve physical fitness and quality of life.[7] Extrapolation of this data is difficult, since mild/moderate HF patients were not tested. This largest and clinically most relevant group may benefit the most. In addition, recent data indicated that women with HF respond differently to interventions than men.[15] Therefore, the primary purpose of this proposal is to compare the 'traditional' moderate-intensity exercise training with the novel high-intensity exercise training in NYHA-class II/III HF patients on physical fitness, clinical outcome and cardiovascular function.

Peripheral factors, such as endothelial dysfunction[8, 16] and skeletal muscle dysfunction, are fundamental to the pathogenesis of HF. Endothelial dysfunction in HF is explained through changes in dilator *and* constricting pathways.[17] A key role is described for nitric oxide (NO), an important dilator substance, to partly explain the endothelial dysfunction in HF patients.[18]

The beneficial effects of physical activity are most likely induced by reversing peripheral limitations such as the elevated vascular tone and endothelial dysfunction.[6, 18] Indeed, in a recent series of experiments, published in Hypertension,[19, 20] I found the fundamental impact of blood flow changes on exercise-induced vascular adaptations (Figure 2).

'Traditional' exercise training exerts its beneficial effects on peripheral vascular function, at least partly, through NO.[8, 18] In addition, no previous study examined the impact of high-intensity training on NO-mediated vascular function. Therefore, the second aim of this proposal is to examine the impact of moderate and high-intensity training in HF on the NO-mediated endothelial function..

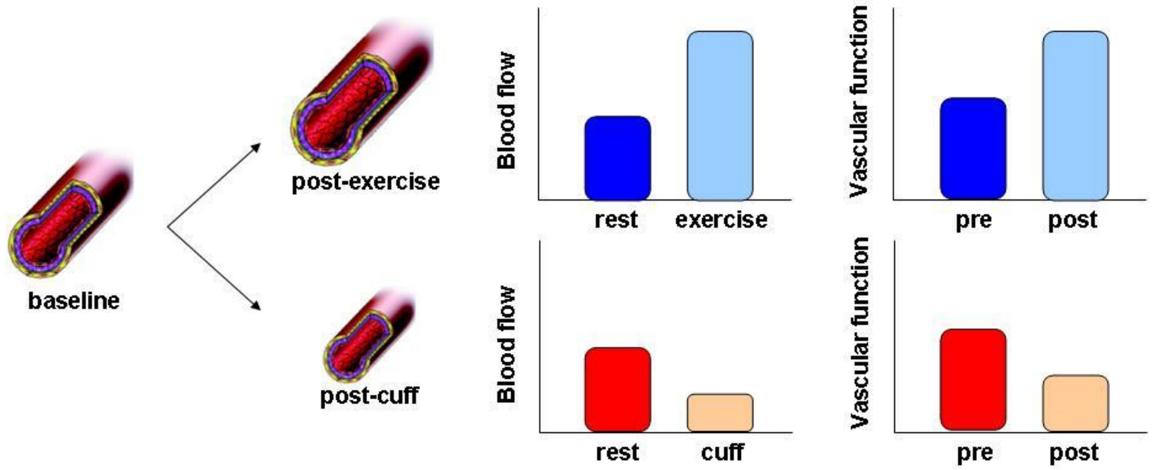

Figure 2. Exercise-induced increase in blood flow improves endothelial function (upper panel), while a decrease in blood flow attenuates endothelial function (lower panel). Adapted from 2 recent papers from Thijssen *et al.* in Hypertension[19, 20]

OBJECTIVES

In general, the objective is to examine the impact of ‘traditional’ moderate and high intensity exercise in heart failure patients (NYHA-class II/III) and elucidate the underlying mechanisms that explain the benefits of these training types. Specifically, we will:

Primary Objective I: Compare the effects of a 12-week ‘traditional’ moderate intensity training program *versus* a novel high intensity exercise training program on physical fitness, clinical outcome and cardiovascular function in HF patients II and III

Primary Objective II: Examine the impact of 12-week moderate *versus* high intensity training in heart failure patients on NO-mediated endothelial function.

We hypothesise that high intensity exercise training in patients with HF will have a superior effect on fitness and outcome compared with moderate intensity exercise training. (AIM I). Moreover, we expect that the larger effect of high intensity training is explained by its larger effect on the NO-mediated endothelial function (AIM II). The results of this study will, therefore, have a major impact on future treatment and prognosis of HF patients.

STUDY DESIGN

50 HF patients (NYHA-class II/III) will be allocated to 12-weeks of; i) moderate intensity exercise training, ii) high intensity exercise training, or iii) control (based on gender and left ventricular ejection fraction) (**Figure 3**). Physical fitness, cardiovascular function, clinical status, and the NO-mediated endothelial function will be examined before and after the intervention (AIM I+II). In addition, the non-invasive FMD-test to examine the NO-mediated endothelial function will also be performed in at week 3, 6 and 9 to examined changes across the 12-week intervention.

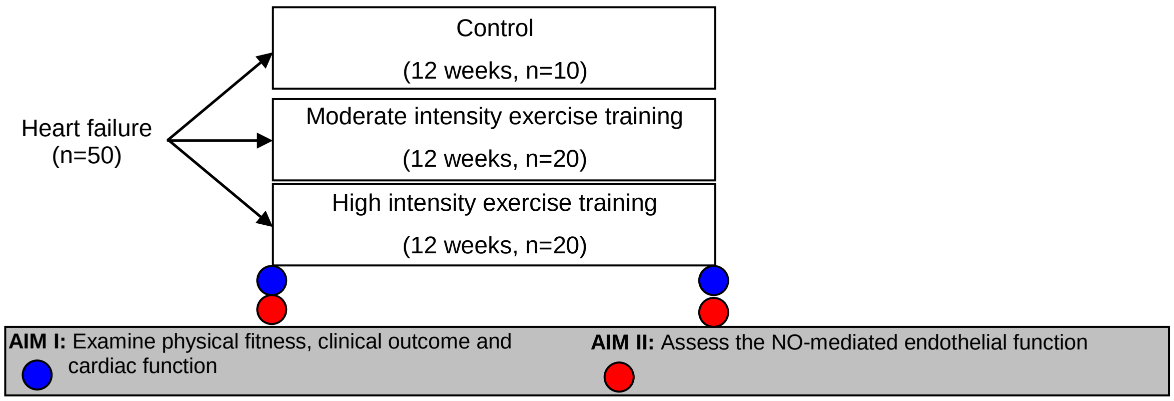

Figure 3. Experimental set-up

Subjects will report to the laboratory on 2 separate days before and after the study. Tests will be performed at the same time of day and under the same conditions (>24 h no exercise, >18 h coffee/tea/chocolate/vitamin C, fasted). In the table below, all tests are summarized in a chronological order.

Table 1. Study design and tests across time.

<u>Before intervention (Day 1, 4 h):</u>

- echocardiography (cardiac function and structure)
- carotid and femoral artery intima-media thickness
- brachial artery NO-dependent endothelium-dependent vasodilation (flow-mediated dilation; 5-min forearm ischemia)
- superficial femoral artery NO-dependent endothelium-dependent vasodilation (flow-mediated dilation; 5-min leg ischemia)
- brachial artery NO-independent endothelium-dependent vasodilation + peak blood flow forearm resistance arteries (5-min ischemic handgrip exercise)
- brachial artery endothelium-independent vasodilation (artery dilation in response to 400 µg sublingual glyceryl trinitrate (GTN))

- maximal cycling test to examine physical fitness

These tests will be repeated after the 12-week intervention. In addition, we will repeat the non-invasive tests performed at Day 1 after 3, 6 and 9 weeks intervention (except the maximal cycling test).

1. STUDY POPULATION

1.1 Population (base)

We will recruit patients with heart failure from the department of Cardiology of the Radboud University Nijmegen Medical Centre (Prof. J Smeets and Dr. A van Dijk). Also, patients will be recruited through advertisements in (regional) news papers and patient associations. And through flyers in health care centers/primary physicians.

1.2 Inclusion criteria

- Heart failure with a NYHA-classification of category II or III
- Ejection fraction of ≤45%
- Patients must be in a stable situation (≥3 month same medication), while efforts are made not to change medication during the study (to minimize the possible effect of medication in the study).

1.3 Exclusion criteria

To exclusively study heart failure, the following subjects will be excluded:

- Type I or II diabetes mellitus
- Severe renal impairment (GFR<30) or proteinuria
- Hepatic impairment
- Hypercholesterolaemia (≥6.5 mmol/L)
- Exercise-induced ischaemia (diagnosed by a cardiologist during a standard cycling test or during the maximal cycling tests as an integral part of the study)
- Hypertension
- Complex ventricular arrhythmias
- Pre-menopausal females or those on hormone replacement therapy
- Pathology/disease that restricts patients from participation to exercise
- Other (serious) pathology, such as chronic obstructive pulmonary disease

1.4 Sample size calculation

To determine the sample size, we have chosen for the change in physical fitness. Previous studies investigating high-intensity training and continuous training included 9-18 participants in each group [7, 21, 22] We therefore propose to include 20 participants per training group, whilst including 10 participants in the control group as the expected differences between the control group and training groups are larger compared to the expected differences between the two training groups.

TREATMENT OF SUBJECTS

Niet van toepassing op dit onderzoek

1.5 Investigational product/treatment

<Please give a description of the intervention (medicinal product, medical device, food supplement, radiation, surgery, behavioural interventions, etcetera). Also use of comparator or placebo should be described.>

Moderate-intensity exercise training.

Exercise training consists of continuous exercise at 60-75% of peak heart rate or peak workload, involving exercise types involving the lower limbs (i.e. walking, cycling). Subjects performed 2 sessions of 45 min for 12 weeks. Exercise intensity will be monitored (Polar®) and maintained continuously at 60-75% of the peak heart rate or peak workload. Workload will be adjusted to meet these criteria. This type of training is typically used in previous studies, including in patients with heart failure.[8, 23]

High-intensity exercise training.

After 10-min warm up at 40% of the peak heart rate or peak workload, subjects perform 10 bouts of 1 min at ~90% peak heart rate or peak workload. This will be separated by 2,5 min at 30-40% peak heart rate or peak workload. Exercise intensity is monitored as described above. Workload (cycling) or speed/inclination (walking) is adjusted to meet these criteria. When subjects use β-blockers, the intensity of the exercise training will be defined by maximal workload. We have extensive experience with both types of exercise.[20, 24-28]

1.6 Use of co-intervention (if applicable)

< Please describe what subjects should do and not do (e.g. use co-medication, adequate contraception, diet). If it is allowed to use co-medication or other kind of intervention, it should be specified on forehand what is allowed.)>

1.7 Escape medication (if applicable)

<Please describe type, dose per unit and maximum dose allowed.>

2. INVESTIGATIONAL MEDICINAL PRODUCT

Not applicable

2.1 Name and description of investigational medicinal product

2.2 Summary of findings from non-clinical studies

<One may refer to the Investigator's Brochure (IB), Investigational Medicinal Product Dossier (IMPD), Summary of Product Characteristics (SPC) or a similar document (if applicable), by mentioning the relevant pages in that document. Be sure that the information is up to date and references to peer reviewed papers in (biomedical/scientific) journals should be given where appropriate.>

2.3 Summary of findings from clinical studies

<See explanatory text of chapter 6.2>

2.4 Summary of known and potential risks and benefits

<See explanatory text of chapter 6.2>

2.5 Description and justification of route of administration and dosage

2.6 Dosages, dosage modifications and method of administration

2.7 Preparation and labelling of Investigational Medicinal Product

2.8 Drug accountability

<Please describe the procedures for the shipment, receipt, disposition, return and destruction of the investigational medicinal products.>

3. METHODS

3.1 Study parameters/endpoints

3.1.1 Main study parameter/endpoint

- Physical fitness
- Brachial artery NO-dependent endothelium-dependent vasodilation.

3.1.2 Secondary study parameters/endpoints (if applicable)

- Cardiac structure and function
- Blood parameters that are (in)directly related to the progression and prognosis of HF

3.1.3 Other study parameters (if applicable)

3.2 Randomisation, blinding and treatment allocation

Subjects will be randomly allocated to the 12-week intervention.

3.3 Study procedures

Before randomisation, subjects will undergo several tests.

<u>Measurements (4 h):</u>

Echocardiography (30 min)

We will examine left ventricular mass (LVM) according to the guidelines of the American Society for Echography. In addition, enddiastolic and endsystolic left ventricular volume will be examined using the biplane Simpson method. This enables us to calculate the ejection fraction (the combination between enddiastolic and endsystolic volumes). Furthermore, using novel echocardiography methods (speckle tracking), we will calculate left and right ventricle strain and strain rate. These techniques are widely applied and can be regarded as the golden standard to measure cardiac function and structure. A well-trained sonographer will perform these tests at the Department of Cardiology. We have a long-standing collaboration with the Department of Cardiology and these measurements of cardiac structure and function are routine in the daily work of the sonographers at the Department of Cardiology.

Physical fitness (60 min)

Physical fitness level will be measured as the peak oxygen uptake during an incremental maximal cycling test (power will be increased with 5-10 W p/m), using a continuous gas analyzer (Oxycon, Jaeger). This test will be performed under supervision of a physician and

an exercise physiologist. Moreover, the test will be performed at the Department of Cardiology at the Radboud University Nijmegen Medical Centre. Although it is not expected that the maximal cycling test can induce a (serious) adverse effect (~1 in 80,000 events), this will ensure a safe environment in which the tests can be performed.

Vascular function and structure (150 min)

Conduit artery diameter and blood flow. Diameter and velocity of conduit arteries (left carotid, bilateral brachial/superficial femoral artery) will be examined using the non-invasive echo-Doppler. This technique is frequently used and widely applied to examine these parameters in the carotid artery. This will also allow measurement of the intima-media thickness (IMT) of these arteries. In addition, by combining the diameter measurements with a pressure signal (non-invasive, derived from a finger cuff from the Portapress), the distensibility or compliance of the artery can be calculated and represents the absolute change in diameter in response to a change in pressure.

Conduit artery endothelium-dependent function. Endothelium-dependent vasodilation of a conduit artery will be examined by inflating a blood pressure cuff around a limb, distal from the imaged artery. Using echo-Doppler, a non-invasive technique to examine the diameter and red blood cell velocity, conduit artery baseline diameter and blood flow will be examined. In addition, baseline blood pressure will be examined. Subsequently, the blood pressure cuff will be inflated to 220 mmHg to block arterial inflow. After 5 minutes, the blood pressure cuff is released and changes in arterial diameter and flow will be assessed. Also blood pressure will be examined. These responses are commonly referred to as flow-mediated dilatation (FDM) and represent a nitric oxide-mediated endothelium-dependent vasodilation in the brachial and superficial femoral artery.[29-34]

Conduit + resistance artery structure. Conduit and resistance artery structure in the arm will be examined by ischemic exercise. After assessment of baseline brachial artery baseline diameter and blood flow, the blood pressure cuffs around the forearm will be inflated to 220 mmHg to block the arterial inflow for 5 minutes. Each individual will perform ischaemic exercise from minute 1 to minute 4. This protocol has recently been demonstrated to result in a peak conduit diameter and also peak blood flow response in brachial artery.[35, 36] In the legs, the cuff will be placed below the imaged artery (because of anatomical reasons it cannot be placed above the imaged artery in analogy with the brachial artery), whilst subjects perform (ischemic) foot flexion exercise from minute 1 till 4.

Conduit artery endothelium-independent function. First, conduit artery baseline diameter and blood flow will be performed using echo-Doppler. After baseline assessment, the endothelium-independent vasodilation will be assessed using a single dose of sublingual

nitroglycerine (0.4 mg). This drug is a nitric oxide donor and can therefore be used to examine the smooth muscle cell sensitivity to NO.

All tests will be performed by a well-experienced sonographer. The Department of Physiology has a long tradition (>10 years) of measuring these vascular characteristics and have even contributed to the development and refinement of the techniques described above. All procedures described above have been applied in numerous previous studies that have been accepted by the ethics committee, whilst we have never experienced any (serious) adverse effects. The latter is in agreement with the general notion and the information from the literature that vascular sonography is a safe technique in humans.

During the intervention at 3, 6, and 9 weeks (2 h):

All measurements listed under *'vascular function and structure'* will be repeated at 3, 6 and 9 weeks to examine the time-course of these vascular adaptations in heart failure patients in response to the different 12-week interventions.

After the intervention:

Repeat of all tests performed on testing days 1 and 2 before the intervention.

3.4 Withdrawal of individual subjects

Subjects can leave the study at any time for any reason if they wish to do so without any consequences. The investigator can decide to withdraw a subject from the study for urgent medical reasons.

3.4.1 Specific criteria for withdrawal (if applicable)

Not applicable.

3.5 Replacement of individual subjects after withdrawal

Based on our experience, we expect a drop out of ~10% in our study. Therefore, we have included 2 additional subjects per study arm. Therefore, we will not replace subjects after withdrawal of the study.

3.6 Follow-up of subjects withdrawn from treatment

We will not follow-up the subjects when withdrawn from the study.

3.7 Premature termination of the study

We do not expect negative consequences in our study (due to our interventions) and have therefore not determined guidelines for premature termination of the study.

4. SAFETY REPORTING

4.1 Section 10 WMO event

In accordance to section 10, subsection 1, of the WMO, the investigator will inform the subjects and the reviewing accredited METC if anything occurs, on the basis of which it appears that the disadvantages of participation may be significantly greater than was foreseen in the research proposal. The study will be suspended pending further review by the accredited METC, except insofar as suspension would jeopardise the subjects' health. The investigator will take care that all subjects are kept informed.

4.2 Adverse and serious adverse events

Adverse events are defined as any undesirable experience occurring to a subject during a clinical trial, whether or not considered related to the investigational drug. All adverse events reported spontaneously by the subject or observed by the investigator or his staff will be recorded.

A serious adverse event is any untoward medical occurrence or effect that at any dose results in death;

- is life threatening (at the time of the event);
- requires hospitalisation or prolongation of existing inpatients' hospitalisation;
- results in persistent or significant disability or incapacity;
- is a congenital anomaly or birth defect;
- is a new event of the trial likely to affect the safety of the subjects, such as an unexpected outcome of an adverse reaction, lack of efficacy of an IMP used for the treatment of a life threatening disease, major safety finding from a newly completed animal study, etc.

All SAEs will be reported to the accredited METC that approved the protocol, according to the requirements of that METC.

4.2.1 Suspected unexpected serious adverse reactions (SUSAR)

Adverse reactions are all untoward and unintended responses to an investigational product related to any dose administered.

Unexpected adverse reactions are adverse reactions, of which the nature, or

severity, is not consistent with the applicable product information (e.g. Investigator's Brochure for an unapproved IMP or Summary of Product Characteristics (SPC) for an authorised medicinal product).

The sponsor will report expedited the following SUSARs to the METC:

- SUSARs that have arisen in the clinical trial that was assessed by the METC;
- SUSARs that have arisen in other clinical trial of the same sponsor and with the same medicinal product, and that could have consequences for the safety of the subjects involved in the clinical trial that was assessed by the METC.

The remaining SUSARs are recorded in an overview list (line-listing) that will be submitted once every half year to the METC. This line-listing provides an overview of all SUSARs from the study medicine, accompanied by a brief report highlighting the main points of concern.

The sponsor will report expedited all SUSARs to the competent authority, the Medicine Evaluation Board and the competent authorities in other Member States.

< *SUSARs that are already reported to the EMEA Eudravigilance database do not have to be once again reported to the competent authority and the MEB because they have direct access to the Eudravigilance database.*>

The expedited reporting will occur not later than 15 days after the sponsor has first knowledge of the adverse reactions. For fatal or life threatening cases the term will be maximal 7 days for a preliminary report with another 8 days for completion of the report.

4.2.2 Annual safety report

In addition to the expedited reporting of SUSARs, the sponsor will submit, once a year throughout the clinical trial, a safety report to the accredited METC, competent authority, Medicine Evaluation Board and competent authorities of the concerned Member States.

This safety report consists of:

- a list of all suspected (unexpected or expected) serious adverse reactions, along with an aggregated summary table of all reported serious adverse reactions, ordered by organ system, per study;

- a report concerning the safety of the subjects, consisting of a complete safety analysis and an evaluation of the balance between the efficacy and the harmfulness of the medicine under investigation.

4.3 Follow-up of adverse events

All adverse events will be followed until they have abated, or until a stable situation has been reached. Depending on the event, follow up may require additional tests or medical procedures as indicated, and/or referral to the general physician or a medical specialist.

4.4 Data Safety Monitoring Board (DSMB)

Currently, relatively little is known about the effects of high-intensity exercise training. We have decided to set-up a DSMB for this study.

STATISTICAL ANALYSIS

4.5 Descriptive statistics

All subject characteristics will be described using descriptive statistics, whilst groups will be compared using ANOVAs.

4.6 Univariate analysis

Group differences at baseline for any of the outcome parameters (primary or secondary) will be examined using a one-way ANOVA with post-hoc t-tests. Also correlation coefficients will be used to examine potential relations between the primary outcome parameters at baseline or after the 12-week intervention

4.7 Multivariate analysis

A 2-way repeated measures ANOVA will be used to examine the changes across the 12-week intervention. Moreover, this analysis also allows examining whether the change in primary/secondary outcome parameter after the 12-week intervention differed between the 3 interventions (moderate intensity training *versus* high intensity training *versus* control). In addition, this analysis also allows correction for covariates that may influence the measurements (e.g. baseline diameter for the FMD-tests).

4.8 Interim analysis (if applicable)

Not applicable

5. ETHICAL CONSIDERATIONS

5.1 Regulation statement

The study will be performed according to the guidelines of the 'Declaration of Helsinki' from 2000 and '*the Medical Research Involving Human Subjects Act (WMO)'*.

5.2 Recruitment and consent

Subjects will be recruited via the Department of Cardiology of the Radboud University Nijmegen Medical Centre (Prof. Dr J. Smeets and Dr A. Van Dijk). Subjects will receive an information letter. After receiving this information sheet, subjects have a 2-week notice to decide whether they like to participate or not. When interested, they can contact the researchers involved in this study for a screening.

5.3 Objection by minors or incapacitated subjects (if applicable)

Not applicable.

5.4 Benefits and risks assessment, group relatedness

Blood will be taken for later analysis. The number of invasive procedures will be minimised to 2.

Exercise training is not associated with a health risk. Moreover, exercise training typically causes a decreased cardiovascular risk, whilst vascular and cardiac function and structure improve after a period of exercise training. Also a number a previous studies have demonstrated that the cardiac workload during high intensity training is not significantly different to the (traditional)moderate-intensity training and can be safely applied in subjects with cardiovascular disease or impaired cardiac function.[9, 13] Some studies have even demonstrated that the beneficial effects of exercise on remodelling of the heart are superior during high-intensity training compared with traditional moderate-intensity training in subjects with coronary artery disease[9] or heart failure[7]. Therefore, both types of exercise are not associated with an increased risk for development of health-related problems. Nonetheless, all exercise sessions will be supervised by a trained exercise physiologists and/or physiotherapist. Moreover, subjects will be monitored using a polar heart rate monitor continuously during every training session, whilst each training will be performed at the hospital. This will ensure that all exercise

training sessions are performed in a well-controlled environment in the unlikely case that something goes wrong before, during or after the exercise session.

Also the non-invasive tests are not related to any potential risk. Although inflation of the blood pressure cuff may induce a slight uncomfortable sensation, this is brief and stops when the cuff is deflated.

At the department of physiology, we have a long-standing tradition in performing all previously mentioned non-invasive (maximal cycling test, cardiac and vascular function and structure) as well as invasive tests (intra-brachial infusion of vasoactive substances). All procedures are performed routinely at the Department of Physiology and have been accepted by the ethics committee in numerous previous applications. Moreover, there is a long history of performing exercise training studies at our Department. Nonetheless, all exercise training sessions and maximal cycling tests will be performed at the hospital and under supervision of highly qualified personnel.

.

5.5 Compensation for injury

Information regarding compensation for potential injury related to the study will be provided in the patient information sheet. This information letter is added as an appendix to this application.

The sponsor/investigator has a liability insurance which is in accordance with article 7, subsection 6 of the WMO.

The sponsor (also) has an insurance which is in accordance with the legal requirements in the Netherlands (Article 7 WMO and the Measure regarding Compulsory Insurance for Clinical Research in Humans of 23th June 2003). This insurance provides cover for damage to research subjects through injury or death caused by the study.

1. € 450.000,-- (i.e. four hundred and fifty thousand Euro) for death or injury for each subject who participates in the Research;
2. € 3.500.000,-- (i.e. three million five hundred thousand Euro) for death or injury for all subjects who participate in the Research;
3. € 5.000.000,-- (i.e. five million Euro) for the total damage incurred by the organisation for all damage disclosed by scientific research for the Sponsor as ‘verrichter’ in the meaning of said Act in each year of insurance coverage.

The insurance applies to the damage that becomes apparent during the study or within 4 years after the end of the study.

5.6 Incentives (if applicable)

6. ADMINISTRATIVE ASPECTS AND PUBLICATION

6.1 Handling and storage of data and documents

Prior to the study, we will set-up a code that will be applied during the entire study. The code will consist of numbers and letters, which eventually leads to a code which cannot be directly linked to the subjects. One of the researchers has a key and will be safeguarded by this researcher.

6.2 Amendments

<*The following text is applicable for studies without an investigational medicinal product*.>
Amendments are changes made to the research after a favourable opinion by the accredited METC has been given. All amendments will be notified to the METC that gave a favourable opinion.

<*The following text is applicable for studies with an investigational medicinal product.*>
A ‘substantial amendment’ is defined as an amendment to the terms of the METC application, or to the protocol or any other supporting documentation, that is likely to affect to a significant degree:

- the safety or physical or mental integrity of the subjects of the trial;
- the scientific value of the trial;
- the conduct or management of the trial; or
- the quality or safety of any intervention used in the trial.

All substantial amendments will be notified to the METC and to the competent authority.

Non-substantial amendments will not be notified to the accredited METC and the competent authority, but will be recorded and filed by the sponsor.
< *Examples of non-substantial amendments are typing errors and administrative changes like changes in names, telephone numbers and other contact details of involved persons mentioned in the submitted study documentation*.>

6.3 Annual progress report

The sponsor/investigator will submit a summary of the progress of the trial to the accredited METC once a year. Information will be provided on the date of inclusion of the first subject, numbers of subjects included and numbers of subjects that have completed the trial, serious adverse events/ serious adverse reactions, other problems, and amendments.

6.4 End of study report

<*The following text is applicable for studies without an investigational medicinal product*.>
The investigator will notify the accredited METC of the end of the study within a period of 8 weeks. The end of the study is defined as the last patient's last visit.

In case the study is ended prematurely, the investigator will notify the accredited METC, including the reasons for the premature termination.

Within one year after the end of the study, the investigator/sponsor will submit a final study report with the results of the study, including any publications/abstracts of the study, to the accredited METC.

<The following text is applicable for studies with an investigational medicinal product.>
The sponsor will notify the accredited METC and the competent authority of the end of the study within a period of 90 days. The end of the study is defined as the last patient's last visit.

In case the study is ended prematurely, the sponsor will notify the accredited METC and the competent authority within 15 days, including the reasons for the premature termination.

Within one year after the end of the study, the investigator/sponsor will submit a final study report with the results of the study, including any publications/abstracts of the study, to the accredited METC and the Competent Authority.

<In case the final study report will not be available within one year, another term should be defined including the reasons.>

6.5 Public disclosure and publication policy

There is no disclosures and/or publication policy.

REFERENCES

1. Ho KK, Pinsky JL, Kannel WB and Levy D. The epidemiology of heart failure: the Framingham Study. *Journal of the American College of Cardiology*. 1993;22:6A-13A.
2. Association AH. *Heart Disease and Stroke Statistics-2003 Update*. 2003;Dallas, Texas: American Heart Association.
3. RIVM. Hartfalen: hoe vaak komt het voor en hoeveel mensen sterven eraan (www.rivm.nl). 2007.
4. Mancini DM, Eisen H, Kussmaul W, Mull R, Edmunds LH, Jr. and Wilson JR. Value of peak exercise oxygen consumption for optimal timing of cardiac transplantation in ambulatory patients with heart failure. *Circulation*. 1991;83:778-86.
5. Myers J, Prakash M, Froelicher V, Do D, Partington S and Atwood JE. Exercise capacity and mortality among men referred for exercise testing. *The New England journal of medicine*. 2002;346:793-801.
6. Maiorana A, O'Driscoll G, Cheetham C, Collis J, Goodman C, Rankin S, Taylor R and Green D. Combined aerobic and resistance exercise training improves functional capacity and strength in CHF. *J Appl Physiol*. 2000;88:1565-70.
7. Wisloff U, Stoylen A, Loennechen JP, Bruvold M, Rognmo O, Haram PM, Tjonna AE, Helgerud J, Slordahl SA, Lee SJ, Videm V, Bye A, Smith GL, Najjar SM, Ellingsen O and Skjaerpe T. Superior cardiovascular effect of aerobic interval training versus moderate continuous training in heart failure patients: a randomized study. *Circulation*. 2007;115:3086-94.
8. Hambrecht R, Fiehn E, Weigl C, Gielen S, Hamann C, Kaiser R, Yu J, Adams V, Niebauer J and Schuler G. Regular physical exercise corrects endothelial dysfunction and improves exercise capacity in patients with chronic heart failure. *Circulation*. 1998;98:2709-15.
9. Rognmo O, Hetland E, Helgerud J, Hoff J and Slordahl SA. High intensity aerobic interval exercise is superior to moderate intensity exercise for increasing aerobic capacity in patients with coronary artery disease. *Eur J Cardiovasc Prev Rehabil*. 2004;11:216-22.
10. Dubach P, Myers J, Dziekan G, Goebbels U, Reinhart W, Muller P, Buser P, Stulz P, Vogt P and Ratti R. Effect of high intensity exercise training on central hemodynamic responses to exercise in men with reduced left ventricular function. *Journal of the American College of Cardiology*. 1997;29:1591-8.
11. Lee IM, Sesso HD, Oguma Y and Paffenbarger RS, Jr. Relative intensity of physical activity and risk of coronary heart disease. *Circulation*. 2003;107:1110-6.
12. Fletcher GF, Balady GJ, Amsterdam EA, Chaitman B, Eckel R, Fleg J, Froelicher VF, Leon AS, Pina IL, Rodney R, Simons-Morton DA, Williams MA and Bazzarre T. Exercise standards for testing and training: a statement for healthcare professionals from the American Heart Association. *Circulation*. 2001;104:1694-740.
13. Meyer K, Samek L, Schwaibold M, Westbrook S, Hajric R, Lehmann M, Essfeld D and Roskamm H. Physical responses to different modes of interval exercise in patients with chronic heart failure--application to exercise training. *European heart journal*. 1996;17:1040-7.
14. Nilsson BB, Westheim A and Risberg MA. Effects of group-based high-intensity aerobic interval training in patients with chronic heart failure. *The American journal of cardiology*. 2008;102:1361-5.
15. Ghanbari H, Dalloul G, Hasan R, Daccarett M, Saba S, David S and Machado C. Effectiveness of Implantable Cardioverter-Defibrillators for the Primary Prevention of Sudden Cardiac Death in Women With Advanced Heart Failure: A Meta-analysis of Randomized Controlled Trials. *Arch Intern Med*. 2009;169(16):1500-06.
16. Kubo SH, Rector TS, Bank AJ, Williams RE and Heifetz SM. Endothelium-dependent vasodilation is attenuated in patients with heart failure. *Circulation*. 1991;84:1589-96.
17. Thijssen DH, Rongen GA, Smits P and Hopman MT. Physical (in)activity and endothelium-derived constricting factors: overlooked adaptations. *The Journal of physiology*. 2008;586:319-24.
18. Maiorana A, O'Driscoll G, Dembo L, Cheetham C, Goodman C, Taylor R and Green D. Effect of aerobic and resistance exercise training on vascular function in heart failure. *American journal of physiology*. 2000;279:H1999-2005.

19. Thijssen DH, Dawson EA, Tinken TM, Cable NT and Green DJ. Retrograde flow and shear rate acutely impair endothelial function in humans. *Hypertension*. 2009;53:986-92.
20. Tinken TM, Thijssen DH, Hopkins N, Black MA, Dawson EA, Minson CT, Newcomer SC, Laughlin MH, Cable NT and Green DJ. Impact of shear rate modulation on vascular function in humans. *Hypertension*. 2009;54:278-85.
21. Fu TC, Wang CH, Lin PS, Hsu CC, Cherng WJ, Huang SC, Liu MH, Chiang CL and Wang JS. Aerobic interval training improves oxygen uptake efficiency by enhancing cerebral and muscular hemodynamics in patients with heart failure. *International journal of cardiology*. 2013;167:41-50.
22. Iellamo F, Caminiti G, Sposato B, Vitale C, Massaro M, Rosano G and Volterrani M. Effect of High-Intensity interval training versus moderate continuous training on 24-h blood pressure profile and insulin resistance in patients with chronic heart failure. *Internal and emergency medicine*. 2013.
23. Hambrecht R, Wolf A, Gielen S, Linke A, Hofer J, Erbs S, Schoene N and Schuler G. Effect of exercise on coronary endothelial function in patients with coronary artery disease. *The New England journal of medicine*. 2000;342:454-60.
24. Black MA, Cable NT, Thijssen DH and Green DJ. Impact of age, sex and exercise on brachial artery flow-mediated dilatation. *American journal of physiology*. 2009;297(3):H1109-16.
25. Thijssen DH, Ellenkamp R, Kooijman M, Pickkers P, Rongen GA, Hopman MT and Smits P. A causal role for endothelin-1 in the vascular adaptation to skeletal muscle deconditioning in spinal cord injury. *Arteriosclerosis, thrombosis, and vascular biology*. 2007;27:325-31.
26. Thijssen DH, Rongen GA, van Dijk A, Smits P and Hopman MT. Enhanced endothelin-1-mediated leg vascular tone in healthy older subjects. *J Appl Physiol*. 2007;103:852-7.
27. Tinken TM, Thijssen DH, Black MA, Cable NT and Green DJ. Time course of change in vasodilator function and capacity in response to exercise training in humans. *The Journal of physiology*. 2008;586:5003-12.
28. Thijssen DH, Dawson EA, Black MA, Hopman MT, Cable NT and Green DJ. Brachial Artery Blood Flow Responses to Different Modalities of Lower Limb Exercise. *Medicine and science in sports and exercise*. 2009;41:1072-79.
29. Mullen MJ, Kharbanda RK, Cross J, Donald AE, Taylor M, Vallance P, Deanfield JE and MacAllister RJ. Heterogenous nature of flow-mediated dilatation in human conduit arteries in vivo: relevance to endothelial dysfunction in hypercholesterolemia. *Circulation research*. 2001;88:145-51.
30. Celermajer DS, Sorensen KE, Gooch VM, Spiegelhalter DJ, Miller OI, Sullivan ID, Lloyd JK and Deanfield JE. Non-invasive detection of endothelial dysfunction in children and adults at risk of atherosclerosis. *Lancet*. 1992;340:1111-5.
31. Kooijman M, Thijssen DH, de Groot PC, Bleeker MW, van Kuppevelt HJ, Green DJ, Rongen GA, Smits P and Hopman MT. Flow-mediated dilatation in the superficial femoral artery is nitric oxide mediated in humans. *The Journal of physiology*. 2008;586:1137-45.
32. Joannides R, Haefeli WE, Linder L, Richard V, Bakkali EH, Thuillez C and Luscher TF. Nitric oxide is responsible for flow-dependent dilatation of human peripheral conduit arteries in vivo. *Circulation*. 1995;91:1314-9.
33. Doshi SN, Naka KK, Payne N, Jones CJ, Ashton M, Lewis MJ and Goodfellow J. Flow-mediated dilatation following wrist and upper arm occlusion in humans: the contribution of nitric oxide. *Clin Sci (Lond)*. 2001;101:629-35.
34. Vita JA and Keaney JF, Jr. Endothelial function: a barometer for cardiovascular risk? *Circulation*. 2002;106:640-2.
35. Black MA, Cable NT, Thijssen DH and Green DJ. Measuring peak conduit artery diameter and peak resistance artery blood flow in the lower limbs using echo-Doppler. *Submitted*. 2009.
36. Naylor LH, Weisbrod CJ, O'Driscoll G and Green DJ. Measuring peripheral resistance and conduit arterial structure in humans using Doppler ultrasound. *J Appl Physiol*. 2005;98:2311-5.
