# Supplementary material for: Effects of High-Intensity Interval Training versus Continuous Training on Physical Fitness, Cardiovascular Function and Quality of Life in Heart Failure Patients
Source: PLoS One. 2015 Oct 30;10(10):e0141256. doi: 10.1371/journal.pone.0141256 (PMC4627811; doi:10.1371/journal.pone.0141256)
Supplement: S1 Table — Data is presented as mean ±SD. †Data missing for 1 control subject. VO2peak; peak oxygen uptake. AT; anaerobic threshold. BA; brachial artery. SFA; superficial femoral artery. GTN; glyceryl trinitrate. CADC; conduit artery dilating capacity. IMT; intima-media thickness. SRAUC; shear rate area-under-the-curve. CADC; conduit artery dilating capacity. LVEDV; left ventricular end-diastolic volume. LVESV; left-ventricular end-systolic volume. IVCT-L/S: isovolumetric contraction time, lateral/septal. IVRT-L/S; isovolumetric relaxation time, lateral/septal. E/A ratio; peak mitral flow velocity during early filling/peak mitral flow velocity during atrial contraction. S/D; systolic flow velocity pulmonary vein/diastolic flow velocity pulmonary vein. E/E’-L/S; peak mitral flow velocity during early filling/peak mitral annulus velocity during early filling, lateral/septal. MLHFQ; Minnesota living with heart failure questionnaire. (DOCX) [file pone.0141256.s003.docx]

**S1 Table.** Results of the control group.

|  | **Control group** |  | |  | |  |
| --- | --- | --- | --- | --- | --- | --- |
| **Physical fitness (n=7)** | *Pre* | | *Post* | | **P-value** | |
| VO_2peak_ (mL/min) | 1363±564 | | 1386±599 | | 0.50 | |
| VO_2peak_ (mL/min/kg) | 17.4±5.8 | | 17.5±5.8 | | 0.79 | |
| VO_2peak_ (% pred. VO_2peak_) | 81±22 | | 82±21 | | 0.74 | |
| Max. workload (Watt) | 103±35 | | 103±41 | | 0.91 | |
| Max. heart rate (/min) | 120±15 | | 120±23 | | 0.97 | |
| VE/VCO_2_ slope | 30.7±7.2 | | 31.4±8.9 | | 0.51 | |
| VO_2_ at AT (mL) | 836±173 | | 914±204 | | 0.08 | |
| Max. O_2_/HR (mL) | 12.6±2.4 | | 12.7±3.2 | | 0.84 | |
|  |  | |  | |  | |
| **Vascular function/structure (n=9)** | *Pre* | | *Post* | |  | |
| BA diameter (mm) | 4.1±0.8 | | 4.0±0.8 | | 0.38 | |
| BA FMD (%) | 5.3±2.0 | | 5.4±2.4 | | 0.83 | |
| BA FMD((%, scaled) | 5.3±1.3 | | 5.3±1.3 | | 0.92 | |
| BA SR_AUC_ (s, 10^3^) | 17.7±13.4 | | 22.5±15.5 | | 0.77 | |
| BA GTN (%) | 13.9±4.0 | | 18.2±6.3 | | 0.07 | |
| BA GTN (%, scaled) | 14.2±3.5 | | 17.8±3.5 | | 0.07 | |
| BA FMD-GTN ratio | 0.39±0.14 | | 0.30±0.08 | | 0.16 | |
| BA CADC (%) | 13.6±5.6 | | 12.1±5.7 | | 0.62 | |
| BA CADC (%, scaled) | 13.3±5.0 | | 12.1±5.0 | | 0.62 | |
| BA peak blood flow (mL/min) | 579±235 | | 654±375 | | 0.43 | |
| SFA diameter (mm) | 6.2±1.0 | | 6.1±1.4 | | 0.68 | |
| SFA FMD (%) | 4.3±1.7 | | 2.5±2.0 | | 0.038 | |
| SFA FMD (%, scaled) | 4.4±1.7 | | 2.5±1.7 | | 0.038 | |
| SFA SR_AUC_ (s, 10^3^) | 11.3±6.7 | | 12.2±7.7 | | 0.65 | |
| CCA IMT (mm) | 0.73±0.13 | | 0.65±0.06 | | 0.31 | |
| CCA IMT-to-lumen ratio | 0.11±0.02 | | 0.11±0.02 | | 0.75 | |
|  |  | |  | |  | |
| **Cardiac function/structure (n=9)** | *Pre* | | *Post* | |  | |
| LVEDV (ml)^†^ | 123±41 | | 131±64 | | 0.53 | |
| LVESV (ml)^†^ | 79±40 | | 81±59 | | 0.58 | |
| Stroke volume (ml)^†^ | 44±9 | | 50±15 | | 0.22 | |
| LVEF (%)^†^ | 38±12 | | 41±13 | | 0.18 | |
| Cardiac output (L/min)^†^ | 2.7±0.6 | | 3.1±1.0 | | 0.14 | |
| Cardiac index ^†^ | 1.4±0.3 | | 1.6±0.5 | | 0.15 | |
| Longitudinal strain | -9±4 | | -10±4 | | 0.61 | |
| Circumferential strain^†^ | -9±3 | | -9±4 | | 0.91 | |
| Radial strain | 22±9 | | 24±10 | | 0.74 | |
| Area strain | -16±6 | | -17±6 | | 0.81 | |
| IVCT-L (ms) | 57±14 | | 54±13 | | 0.19 | |
| IVCT-S (ms) | 63±14 | | 57±8 | | 0.10 | |
| IVRT-L (ms) | 158±46 | | 187±38 | | 0.16 | |
| IVRT-S (ms) | 187±38 | | 176±27 | | 0.48 | |
| E/A | 0.96±0.55 | | 0.98±0.63 | | 0.68 | |
| S/D | 1.29±0.36 | | 1.58±0.27 | | 0.08 | |
| E/E’-L | 8.7±3.9 | | 12.9±12.1 | | 0.015 | |
| E/E’-S | 8.8±2.2 | | 12.5±8.6 | | 0.11 | |
|  |  | |  | |  | |
| **SF-36 (n=5)** | *Pre* | | *Post* | |  | |
| Physical functioning | 66±20 | | 59±21 | | 0.08 | |
| Physical health subscore^†^ | 76±8 | | 71±7 | | 0.07 | |
| Mental health subscore^†^ | 91±6 | | 88±2 | | 0.47 | |
| Total score^†^ | 81±7 | | 77±5 | | 0.07 | |
|  |  | |  | |  | |
| **MLHFQ (n=7)** | *Pre* | | *Post* | |  | |
| Total score | 8±8 | | 8±7 | | 0.96 | |

Data is presented as mean ±SD. ^†^Data missing for 1 control subject. VO_2peak_; peak oxygen uptake. AT; anaerobic threshold. BA; brachial artery. SFA; superficial femoral artery. GTN; glyceryl trinitrate. CADC; conduit artery dilating capacity. IMT; intima-media thickness. SR_AUC_; shear rate area-under-the-curve. CADC; conduit artery dilating capacity. LVEDV; left ventricular end-diastolic volume. LVESV; left-ventricular end-systolic volume. IVCT-L/S: isovolumetric contraction time, lateral/septal. IVRT-L/S; isovolumetric relaxation time, lateral/septal. E/A ratio; peak mitral flow velocity during early filling/peak mitral flow velocity during atrial contraction. S/D; systolic flow velocity pulmonary vein/diastolic flow velocity pulmonary vein. E/E’-L/S; peak mitral flow velocity during early filling/peak mitral annulus velocity during early filling, lateral/septal. MLHFQ; Minnesota living with heart failure questionnaire.
